# Nerve-Shattered Soldiers and Their Treatment

**Published:** 1917-09-15

**Authors:** 


### NERVE-SHATTERED SOLDIERS AND THEIR TREATMENT

### Dr. Lumsden's Excellent Scheme.

A VERY sensible suggestion has been made by Dr. Lumsden in the *Times*. It is that soldiers suffering from functional and curable nervous disease, disease that is eminently amenable to treatment, but to which treatment in hospital is not appropriate and not likely to be successful, should be "boarded out" in country homes, in gardeners' cottages, on farms, and in similar places. The suggestion is a good one for many reasons. These cases, as has been said, are not suitable for hospital treatment. The hospital atmosphere, the atmosphere of invalidism, sickness, and incapacity, is not good for them. Their minds are in a morbid state, they are introspective and amenable to suggestion, and are very likely to reproduce the symptoms that they witness exhibited by those around them. They reinforce and corroborate one another's feelings. The hospital is almost a forcing house of symptoms. It is important for their recovery that they should be separated from other patients suffering in the same way, and should live amongst healthy people. At the same time, their own homes are by no means appropriate places for such patients. Here they are the objects of too much solicitude, and receive too much sympathy and assistance; and thus form habits that tend rather to the confirmation and fixation of the nervous condition than to its recovery. The best human surroundings for them are not those of invalids like themselves, or those of lavish interest and sympathy which increase their tendency to self-absorption, to introspection, to attention to their own symptoms, and to self-pity and self-interest, but rather the society of others who are too busy to make a study of them, of others who are absorbed in their own occupations, and whose interests are externalised on busy industry.

### A NEW ASPECT OF LIFE.

Great is the force of example. Especially great is it to the impressible, such as for the most part these patients are. If they are removed from the aimless and idle life of the hospital to surroundings in which every hand but their own is at work, and every interest but their own is thrown outward upon work to be done, their own interests can scarcely fail to be drawn out of themselves. Moreover, to very many of them there will be the interest of novelty. They have most of them been town-dwellers, and have never known the varied aspects of nature or the varied interests of country life. They will be introduced into a wider and more spacious horizon. They will view life from a new aspect. They will be tempted out of doors. Simple jobs within their capacities, interesting work, novel in kind, can be found for them. All the conditions of their lives will be curative.

The scheme is to include, according to Dr. Lumsden, the following features:

1. The patient is to be freed completely from the brooding horror of returning to the circumstances that have brought him to this pass. He is to be assured that his connection with the Army is for ever broken.

2. The host who receives him incurs no responsibility for his health or recovery, but merely carries out instructions, which are of a simple character.

3. The pension is to be temporary, and may be reduced if it appears that the pensioner is not using every effort to get well.

4. He will receive his pension, but no pay for the work he may do. We are not at all sure that this is the best arrangement. Every possible incentive should be held out to the patient to put forth every effort for his own recovery, and there is little incentive to work if the work is unremunerated. That it should not be remunerated at its full value, we should agree; but we think it should receive some little remuneration calculated on its value. If this is not done, the pensioner will be apt to think that he is being exploited, a very unreasonable suspicion, no doubt, but such patients are apt to be suspicious; and the working classes are just now full of suspicion that they are being exploited by a treacherous and malignant monster called Capital.

5. Full instructions are to be sent to the hosts to how the pensioner is to be treated, and both pensioner and host are to report progress once a month. We think this is not often enough. It should be once a fortnight, for the treatment is to last only three months. This period, we think, should be made elastic. Probably it will seldom be able to be diminished, but it may need in some cases to be increased in order to obviate the danger of a relapse. These, however, are details that can easily be adjusted.

The scheme strikes us as on the whole an excellent one, and in view of the wonderful sympathy and help that have been manifested on all sides and from all classes, and especially from the wealthy, towards our wounded and incapacitated soldiers, we share to the full Dr. Lumsden's confidence that hosts in amply sufficient numbers will offer the necessary accommodation and care. We wish the scheme all the success it deserves.

An alternative scheme has been suggested by another correspondent of the *Times*, a scheme by which a house capable of accommodating twelve officers and 250 men, should be taken over for the purpose of receiving these shell-shocked sufferers. The house has been carefully inspected, approved, and recommended. All that is wanting is the consent of the owner, and the *Times* correspondent is indignant and suggests that the owner's opposition shall be set aside and the house commandeered. No doubt this would be justifiable if the purpose were adequate, but such an institution, with 262 patients all suffering from very much the same complaints, would be open to all the objections that are enumerated above, and would be, if not the very worst place, still very far from the best place for their treatment. The ideal mode of treatment is by the scheme of Dr. Lumsden.

